# Endovascular management of intracranial aneurysms at Chris Hani Baragwanath Academic Hospital

**DOI:** 10.4102/sajr.v27i1.2634

**Published:** 2023-05-26

**Authors:** Herchel Clarke, Trevor Nefale, Victor Mngomezulu

**Affiliations:** 1 Department of Radiology, Faculty of Health Sciences, University of the Witwatersrand, Johannesburg, South Africa

**Keywords:** endovascular management, intracranial aneurysms, cerebral aneurysms, interventional neuroradiology, cerebral angiography

## Abstract

**Background:**

Worldwide, intracranial aneurysms are associated with a high mortality rate. While endovascular management has proven to be the choice of treatment in selected patients, patient demographics and aneurysm characteristics differ between study populations.

**Objectives:**

This study aimed to investigate the profile of patients with intracranial aneurysms who underwent endovascular management in the Interventional Neuroradiology Unit at Chris Hani Baragwanath Academic Hospital. Patient demographics, risk factors, indications, aneurysm characteristics and intra-operative complications were studied.

**Method:**

This was a 3-year retrospective study of all adult patients between 01 January 2018 and 31 January 2021. The Chi-square test was used to compare categorical variables.

**Results:**

A total of 77 patients were included in this study. The mean age of the patients was 47 ± 11.6 with a male-to-female ratio of 1:1.8. Hypertension was the most reported risk factor in 27% of patients. There was no statistical correlation between the gender groups according to presentation, multiplicity, aneurysmal size dimensions and locations. According to the presentation, there was statistical significance in ruptured intracranial aneurysms (*p* = 0.020), neck size dimensions less than 4 mm (*p* = 0.010), and aneurysms located in the internal cerebral artery (ICA) circulation (*p* = 0.001).

**Conclusion:**

The study findings support known parameters including females and anterior circulation aneurysm preponderance, and the low complication risk of endovascular management. Interestingly, intracranial aneurysms presented with rupture at smaller size dimensions.

**Contribution:**

This study provides valuable insights into intracranial aneurysm characteristics and endovascular management efficacy in a resource-limited setting.

## Introduction

Intracranial aneurysms are a common condition affecting nearly 3.2% of the adult (mean age of 50 years) population globally.^1,2^ Unruptured aneurysms constitute 2% – 5% of the general population.^3^ Ruptured intracranial aneurysms with subarachnoid haemorrhage (SAH) have a significantly poor outcome with a mortality rate as high as 20% – 45% and a morbidity rate of approximately 30% – 40%.^4,5^ According to the International Study of Unruptured Intracranial Aneurysms (ISUIA) trial, unruptured aneurysms reportedly rupture at a minimal annual rate of approximately 0.8%.^6^

According to the World Health Organization (WHO), non-communicable diseases (NCDs) are the leading cause of death globally, with low- and middle-income countries carrying the highest burden,^7,8,9^ thus harbouring the risk factors for developing intracranial aneurysms. Several risk factors have been linked to the development of intracranial aneurysms such as female predilection, hypertension, smoking, high alcohol consumption, familial or genetic syndromes and certain aneurysmal morphological features.^1,2,4^ These risk factors are not universal as there remains variation in the literature across demographics. Intracranial aneurysms in pregnancy are reported to occur in approximately 1.8% of women, with rupture occurring in the range of 1–10 per 100 000 pregnancies.^10,11^ Furthermore, human immunodeficiency virus (HIV) infection remains a leading cause of mortality and morbidity in South Africa.^8^ In a local study, HIV-associated intracranial aneurysms were shown to occur in patients at a younger age and often with complex morphological features.^12^ The prevalence of multiple intracranial aneurysms is 17% – 33.4% in patients suffering from subarachnoid haemorrhage.^13^ Multiple aneurysms as a risk factor are, however, not yet well understood. A South African audit found that patients with multiple aneurysms had a predominance for patients older than 40 years.^14^

Intracranial aneurysm rupture depends on certain characteristics such as size, location and shape.^15^ Intracranial aneurysms are reported to have an 85% anterior circulation predominance.^5^ In an African study, variations in the location of aneurysms differed in population groups.^16^ Aneurysm size categorisation into small, medium and large differs in the literature and is also controversial in the decision to treat; several references refer to an aneurysm size of 7 mm as one of the criteria for treatment.^1,17,18,19^ International literature recommends intervention only for aneurysms greater than 7 mm or aneurysms in the posterior circulation; however, in a South African study, it was found that ruptured aneurysms occurred with aneurysm sizes less than 7 mm.^18^ The mean aneurysm size dimensions also appeared to rupture at smaller sizes in the HIV-infected population.^12^

Endovascular management of intracranial aneurysms is an evolving field with new technological advancements.^20^ Endovascular coiling of intracranial aneurysms has been shown to significantly improve outcomes compared with surgical clipping.^15,17,19,21,22^ In a recent meta-analysis, surgical clipping attracted a poorer outcome compared with endovascular management; no significant difference was observed in mortality and re-bleeding.^23^ Although endovascular management is becoming more widely adopted as the first-line management of ruptured and unruptured aneurysms, the scope and implementation in Africa are still considered suboptimal.^24,25^ In a South African-based study, a 16% reduction in mortality and major morbidity was achieved through endovascular management.^26^

Endovascular coiling was also shown to be a more durable treatment method compared with surgical clipping in terms of patients’ long-term outcomes.^27^ Thrombo-embolic events and intra-operative rupture are the most common complications experienced with endovascular coiling of intracranial aneurysms.^24,28^ In a recent multicentre cohort, thrombo-embolic events occurred more frequently, with a female and middle cerebral artery (MCA) predominance. In addition, it was found that aneurysms that are small and located in the anterior cerebral and communicating arteries were more frequently associated with intraoperative rupture.^24,28^

This study aimed at assessing the profile of patients with intracranial aneurysms who underwent endovascular management at Chris Hani Baragwanath Academic Hospital (CHBAH) in Soweto, South Africa, investigating the patient demographics, risk factors, aneurysm characteristics and intraoperative complications.

## Materials and methods

This was a retrospective study of patients who underwent endovascular management of ruptured and unruptured intracranial aneurysms in the Interventional Neuroradiology Unit at CHBAH over a period of 3 years, from 01 January 2018 to 31 January 2021. The data were recorded on an electronic data recording sheet, and included patient demographics (age and gender), risk factors (hypertension, hypercholestrolaemia, smoking, family history, genetics and pregnancy), presentation on admission (ruptured or unruptured), aneurysm location (anterior cerebral artery [ACA], anterior communicating artery [AComA], posterior cerebral artery [PCA], posterior communicating artery [PComA], middle cerebral artery [MCA], internal cerebral artery [ICA] and basilar tip [BT]), aneurysm size (neck and maximum diameter dimensions), aneurysm multiplicity (single or multiple), and intraoperative complications (aneurysm perforation and thromboembolic event). Aneurysm size definitions were adopted from Pierot et al. where maximum diameter (dome width) was dichotomised into < 5 mm and ≥ 5 mm and a neck size ≥ 4 mm was defined as a wide-neck aneurysm, rendering a narrow-neck aneurysm as < 4 mm.

All patients 19 years of age or older who underwent endovascular management for index ruptured or unruptured intracranial aneurysms with complete records (images and angiogram reports) were included. Angiogram reports and accompanying images were obtained from the Picture Archiving and Communication System (PACS) in the Radiology Department at CHBAH. The principal investigator was primarily responsible for data collection and data analysis supported by a biostatistician. A *p*-value < 0.05 was used to determine statistical significance by utilising the Chi-square test.

### Ethical considerations

The study was approved by the Human Research Ethics Committee of the University of the Witwatersrand (certificate number M211038). Participants’ consent was not sought as this was a retrospective record review and to maintain strict anonymity, no personally identifiable information was recorded.

## Results

A final sample of 77 patients who underwent endovascular management for intracranial aneurysms were included. A total of 49 were female and 28 were male patients (1:1.8 male:female ratio) with a mean age of 47 years (*p* = 0.217). The angiogram reports yielded 21 (27%) patients with documented hypertension, one patient with hypercholesterolaemia (1%), and two (4%) patients from the female group who presented with ruptured intracranial aneurysms during pregnancy.

Sixty six patients (87%) presented with ruptured intracranial aneurysms. One patient’s mode of presentation was unspecified on the angiogram report and there was no supporting imaging on PACS as the patient was referred from a regional hospital; therefore, the patient was excluded from the statistical analysis according to presentation. A total of 89 aneurysms were detected among the 77 patients. The majority of patients had a single aneurysm (88%), while nine (12%) presented with multiple aneurysms. Most intracranial aneurysms were narrow neck aneurysms (83%). Similarly, the maximum diameter dimensions were less than 5 mm in 83%. There was no statistical significance in presentation, multiplicity, neck and maximum diameter dimensions regardless of gender (Table 1).

**TABLE 1:** Intracranial aneurysm characteristics according to gender.

Aneurysm characteristics	Male	Female	Total		*p*
			*n*	%	
**Presentation†**	-	-	-	-	0.270
Unruptured	2	8	10	13	-
Ruptured	25	41	66	87	-
**Multiplicity**	-	-	-	-	0.591
Single	24	44	68	88	-
Multiple	4	5	9	12	-
**Neck (mm)**	-	-	-	-	0.875
< 4	28	46	74	83	-
> 4	6	9	15	17	-
**Maximum diameter (mm)**	-	-	-	-	0.974
< 5	24	39	63	71	-
> 5	10	16	26	29	-

† , Excluded one unspecified internal cerebral artery aneurysm presentation.

There was no statistical significance in presentation regardless of aneurysm multiplicity and maximum diameter dimensions. Narrow neck aneurysms (< 4 mm) constituted a significant proportion (83%) of aneurysms on presentation (*p* = 0.01) (Table 2).

**TABLE 2:** Characteristics of treated intracranial aneurysms.

Aneurysm characteristics	Presentation†				
	Ruptured	Unruptured	Total		*p*
			*n*	%	
**Multiplicity**	-	-	-	-	0.097
Single	59	7	66	87	-
Multiple	7	3	10	13	-
**Neck (mm)**	-	-	-	-	0.010
< 4	58	6	64	82	-
> 4	9	5	14	18	-
**Maximum diameter (mm)**	-	-	-	-	0.254
< 5	48	6	54	70	-
> 5	19	5	24	30	-

† , Excluded one unspecified mode of presentation.

In total, AComA (22%) aneurysms were the most common. Basilar tip aneurysms (2%) accounted for the least common location. Males were mostly affected by ICA (26%) and AComA (24%) aneurysms. Anterior cerebral artery (22%) and AComA (22%) aneurysm locations were equally distributed among females. No statistical significance was demonstrated in intracranial aneurysm location according to gender (Table 3).

**TABLE 3:** Location of intracranial aneurysms according to gender.

Location	Male (*n* = 34)	Female (*n* = 55)	Total (*n* = 89)		*p*
			*n*	%	
ACA	6	12	18	20	0.634
AComA	8	12	20	22	0.851
PCA	1	4	5	6	0.389
PComA	3	10	13	15	0.389
MCA	6	8	14	16	0.696
ICA	9	8	17	19	0.164
BT	1	1	2	2	0.728

ACA, anterior cerebral artery; AComA, anterior communicating artery; PComA, posterior communicating artery; PCA, posterior cerebral artery; MCA, middle cerebral artery; ICA, internal carotid artery; BT, basilar tip.

In total, 88% of the study population presented with ruptured intracranial aneurysms (*p* = 0.02) and of these, AComA aneurysms were the most common (23%). All patients who underwent intervention for AComA aneurysms were ruptured on presentation; however, there was no statistical significance according to location (*p* = 0.064). The ICA aneurysms demonstrated statistical significance (*p* = 0.001) according to location (Table 4).

**TABLE 4:** Location of intracranial aneurysms according to presentation.

Location	Presentation†				
	Unruptured (*n* = 10)	Ruptured (*n* = 72)	Total (*n* = 82)		*p*
			*n*	%	
ACA	1	16	17	21	0.352
AComA	0	19	19	23	0.064
PCA	1	4	5	6	0.582
PComA	2	11	13	16	0.701
MCA	1	13	14	17	0.526
ICA†	5	7	12	15	0.001
BT	0	2	2	2	0.594

ACA, anterior cerebral artery; AComA, anterior communicating artery; PComA, posterior communicating artery; PCA, posterior cerebral artery; MCA, middle cerebral artery; ICA, internal carotid artery; BT, basilar tip.

† , Excluded one unspecified ICA aneurysm presentation.

Referring to Table 5, there were three patients who developed intraoperative complications from the total of 82 aneurysms managed endovascularly, yielding a complication rate of approximately 4%. In all three instances, the complications were immediately identified and managed. Two patients presented with no immediate postoperative neurology. The third patient had no records available regarding the immediate neurological status of the patient in the postoperative angiogram report.

**TABLE 5:** Intraoperative complication, patient demographics and aneurysmal characteristics.

Complication	Patient 1: Aneurysm perforation	Patient 2: Thrombo-embolic event	Patient 3: Thrombo-embolic event
Gender	F	F	F
Age	49	25	59
Risk factors	Hypertension	Pregnancy	Unspecified
Presentation	Ruptured	Ruptured	Unruptured
Location	AComA	ICA	ICA
Neck size (mm)	< 4	< 4	> 4
Maximum diameter size (mm)	< 5	< 5	< 5

F, female; AComA, anterior communicating artery; ICA, internal carotid artery.

## Discussion

Consistent with local and international studies, this South African-based population study of patients who underwent endovascular management for intracranial aneurysms demonstrated a female predominance with a male: female ratio of 1:1.8.^2,11,14,16,18,21,25^ There was poor yield of patient risk factors in the angiogram reports. Hypertension was the most reported risk factor consistent with the literature.^2,4,15^ Intracranial aneurysms in pregnancy are rare and it is postulated that normal haemodynamic changes during pregnancy may play a role in the risk for complications in patients harbouring intracranial aneurysms.^11^ Although not included in this study as a risk factor, HIV-associated aneurysms have been shown to present in younger patients and at smaller sizes.^12^

There is a high population variance regarding intracranial aneurysm location in the literature, some with a posterior circulation predominance and some with an anterior predominance.^14,15,16,23,26^ This study population demonstrated an AComA predominance, regardless of gender or presentation. Internal cerebral artery aneurysms demonstrated significance according to presentation and had a near equal distribution between the ruptured and unruptured groups.

Patients with ruptured aneurysms often present with subarachnoid haemorrhage on initial diagnostic imaging.^2^ A patient presenting with an unruptured aneurysm may be asymptomatic or present with isolated cranial nerve palsies or with non-specific symptoms such as headaches. There was a statistical difference between patients presenting with ruptured intracranial aneurysms on presentation in this study. This is consistent with the literature where it has been shown in a systematic review and meta-analysis that the prevalence of unruptured intracranial aneurysms ranged between 0% and 41.8% with an overall mean prevalence of 2.8% between studies.^29^

According to the literature, larger aneurysms are an independent risk factor for the risk to rupture. Although the literature is not clear as to the exact dimensions, some studies have reported maximum aneurysm diameters of 6 mm – 7 mm as a guide for the decision to treat.^1,17,18,19^ This study demonstrated that most intracranial aneurysms presenting with rupture were less than 4 mm and less than 5 mm for aneurysmal neck and maximum diameter size dimensions, respectively. Although there are known independent risk factors increasing the risk of developing intracranial aneurysms, because of the poor reporting yield in angiogram reports in this study, comparisons could not be sought.

Endovascular neuro-intervention has been shown to reduce morbidity and, in some studies mortality, compared to surgical clipping.^19,21,23,24^ The two most common complications in endovascular neuro-intervention are perioperative intracranial aneurysm perforation and acute thromboembolic phenomenon. This study population had a 4% complication rate that correlates with the literature documenting 2% – 8%, while surgical clipping complications can range between 15% and 50%.^28^ In this study, endovascular intervention for intracranial aneurysms showed a low complication risk and was managed actively to minimise postoperative long-term complications.

### Limitations

This was a retrospective, single institutional study with a small sample size limiting comparison of the location, aneurysm morphology and larger-sized aneurysm subcategories. In addition, angiogram reports did not include comprehensive documentation of patient risk factors and were primarily dependent on information provided on the patient’s request forms for endovascular management. Finally, this study did not investigate long-term clinical outcomes on subsequent follow-up cerebral angiography.

## Conclusion

Overall, this study supports known parameters including a female and anterior circulation aneurysm preponderance, and the low complication risk of endovascular management. Interestingly, intracranial aneurysms presented with rupture at smaller size dimensions. Multi-institutional prospective studies are recommended to further investigate aneurysm morphology and risk factor stratification. The findings may influence the decision threshold to treat smaller-sized intracranial aneurysm dimensions and anterior circulation aneurysms in our population.
